# Muscle synergies are shared across fundamental subtasks in complex movements of skateboarding

**DOI:** 10.1038/s41598-024-63640-5

**Published:** 2024-06-04

**Authors:** Paul Kaufmann, Lorenz Zweier, Arnold Baca, Hans Kainz

**Affiliations:** 1 Department of Biomechanics, Kinesiology and Computer Science in Sport, Centre for Sport Science and University Sports, University of Vienna, Auf der Schmelz 6a (USZ II), 1150 Vienna, Austria; 2 Neuromechanics Research Group, Centre for Sport Science and University Sports, University of Vienna, Vienna, Austria

**Keywords:** Motor control, Neurophysiology, Learning and memory

## Abstract

A common theory of motor control posits that movement is controlled by muscle synergies. However, the behavior of these synergies during highly complex movements remains largely unexplored. Skateboarding is a hardly researched sport that requires rapid motor control to perform tricks. The objectives of this study were to investigate three key areas: (i) whether motor complexity differs between skateboard tricks, (ii) the inter-participant variability in synergies, and (iii) whether synergies are shared between different tricks. Electromyography data from eight muscles per leg were collected from seven experienced skateboarders performing three different tricks (Ollie, Kickflip, 360°-flip). Synergies were extracted using non-negative matrix factorization. The number of synergies (NoS) was determined using two criteria based on the total variance accounted for (tVAF > 90% and adding an additional synergy does not increase tVAF > 1%). In summary: (i) NoS and tVAF did not significantly differ between tricks, indicating similar motor complexity. (ii) High inter-participant variability exists across participants, potentially caused by the low number of constraints given to perform the tricks. (iii) Shared synergies were observed in every comparison of two tricks. Furthermore, each participant exhibited at least one synergy vector, which corresponds to the fundamental ‘jumping’ task, that was shared through all three tricks.

## Introduction

The human body is a redundant system with an infinite number of degrees of freedom. Understanding how the central nervous system solves the redundancy problem of the musculoskeletal system and controls human movements is challenging. One common theory of motor control posits that the central nervous system coordinates muscles by activating a limited number of pre-established sets of muscles, called synergies, rather than controlling each muscle independently^1–4^. Each synergy comprises multiple muscles, which can be activated by a single activation command, thereby reducing the complexity of motor control^5^. Put simply, muscle synergies are a group of co-active muscles, termed synergy vectors (W), which are recruited by an activation coefficient (C), corresponding to a single control input^2,6^.

To gain insight into human motor control, muscle synergies can be extracted from electromyography (EMG) recordings of multiple muscles, using factorization methods^7^. These provide unique insights into healthy and pathological motor control. A commonly used measure to assess the complexity of motor control is the total variance in muscle activity accounted for (tVAF) by a given number of synergies. It has been demonstrated that tVAF is higher in children with cerebral palsy compared to typically developing children, indicating that fewer synergies and therefore a simpler motor control is required for cerebral palsy gait^8^. Similarly, after stroke, a higher tVAF and a decreased number of synergies was observed during walking compared to unimpaired individuals^9^. In healthy cyclists, it has been shown that the tVAF remains largely unchanged when different constraints were applied, such as seated versus standing cycling or submaximal versus maximal effort^10^. A comparison between experienced rowers and untrained individuals did not reveal any difference in the tVAF, indicating that rowing expertise does not necessitate the development of novel synergies^11^. These findings indicate that a lower tVAF is associated with a more complex motor control, and that the tVAF is consistent across movements with different constraints. However, most studies related to physical activities included daily and mainly cyclic movements. Therefore, it is currently unclear whether the tVAF and number of synergies change with an increasing difficulty of movements.

Previous studies have identified similar synergies across different movements. These so-called shared synergies describe similar movement fragments, which correspond to physical subtasks with the same mechanical goals. In contrast, movement-specific subtasks with different functions are activated by task-specific synergies^12^. As an example, neither tVAF nor the number of needed synergies differed between Nordic-walking and conventional walking, and all synergies except one were shared between both tasks. The one task-specific synergy was responsible for the upper limb movement, while the shared synergies mainly activated trunk and lower limb muscles. This outlines a high similarity in motor control among two similar movements^13^. Furthermore, several studies by Allen et al.^14–16^ showed that the number of shared synergies between standing reactive balance tasks and walking depended on the participants’ movement proficiency. In detail, the number of shared synergies, was higher after a dance based rehabilitation in people with Parkinson’s disease^14^, in a control group compared with post-stroke patients^15^, and in expert dancers versus novices^16^. Additionally, shared synergies were found between walking and cycling^17^, walking and slipping^12^, and stepping and non-stepping postural behaviors^18^.

Analyses of daily and automated tasks, such as walking, may not necessarily translate to an understanding of more complex movements with a lower level of expertise. Wulf and Shea^19^ proposed that a comprehensive understanding of motor learning requires insights into skill acquisition beyond those acquired within a single learning session or where participants have a high level of expertise. Consequently, the analyses of muscle synergies in movements that have been acquired only over months to years of training, where not every attempt leads to success, could not only enhance our comprehension of motor control in these tasks but also contribute to a deeper understanding of movement learning in general. Movement learning is of significant relevance in rehabilitation, especially for patients with neurological impairments who must re-learn fundamental tasks like walking. In this context, recent studies have extensively examined motor complexity, inter-participant variability, and synergy similarities among tasks in stroke survivors^9,15,20–22^. However, to the best of our knowledge, no studies have investigated the presence of shared synergies between movements with a high level of complexity requiring high memory and processing demands. Additionally, only one study has explored inter-participant synergy similarities in a complex movement^23^. This study demonstrated the presence of consistent muscle synergies among trained gymnasts performing a backward giant swing, a motor task necessitating months to years of consistent training.

The main objective of this study was to enhance our understanding of neuromuscular control in motor tasks that require months to years of dedicated training and even then, might not be fully automated. For this purpose, skateboarding, a sport that was introduced to the Olympic Games in 2020, was investigated. Several biomechanical studies on skateboarding analyzed the kinetics of jumping and landing during tricks to gain insights for injury prevention and equipment development^24–27^. Furthermore, a recent study employed inertial measurement units to identify the key temporal events during a jump with the skateboard^28^. There is a paucity of research investigating muscle activity and motor control during skateboarding. In a conference abstract, Crockett & Jensen^29^ demonstrated that the tibialis anterior and the rectus femoris muscles are the primary active muscles during a so-called Ollie, which is a fundamental jump with the skateboard. In a study by Vorlíček et al.^30^, EMG signals were compared between an Ollie and a switch-stance Ollie. The authors identified slight difference in muscle activity, which they attributed to the less common practice of the switch-stance compared to the standard Ollie. Cesari et al.^31^ analyzed the ability to evoke muscle activities of a skateboard trick, simply by listening to the sound of the trick. To the best of our knowledge, there are no studies examining whether synergies vary among skateboarders and between different tricks. Addressing this gap in knowledge will not only enhance our understanding of skateboarding but also contribute to our fundamental knowledge of how the central nervous system controls complex movements that require high memory and processing demands.

Our aims were to (i) ascertain whether the number of synergies and the tVAF differ between skateboard tricks, (ii) quantify the inter-participant variability in synergies, and (iii) evaluate whether synergies are shared between different tricks. We hypothesize that (i) the number of synergies increases with the difficulty of the trick, (ii) synergies are consistent across participants, and (iii) some but not all synergies are shared between different tricks.

## Materials and methods

### Participants

Seven male, healthy skateboarders (age: 29.71 ± 4.58 years; body weight: 75.43 ± 9.07 kg) with at least 7 years of skateboarding experience volunteered to participate in the current study. All participants were recreational skateboarders who occasionally participated in local competitions. None of them had sustained any lower limb injuries within the six months preceding the data collection. The study was approved by the ethics committee of the University of Vienna (reference number: 00621) and all aspects of the study were conducted in agreement with the relevant guidelines and regulations. Prior to data collection, all participants provided written informed consent.

### Experimental setup and data collection

In order to replicate skateboard tricks in a laboratory setting that resembles typical skate environments, a skateable track comprising four wooden panels (each 210*65*2 cm) was placed on the floor. These panels were arranged in such a way that a preparation space (2 panels in a row) was created for gaining speed and preparing for the trick, while an execution space (2 panels side by side) was established for performing the trick and landing. Each participant had 10 to 20 min time to warm up on this setup. Subsequently, a cluster of three markers was attached to each participant’s sacrum. The marker trajectories were captured and used to time normalize the movement, as described in the next section. Following shaving and cleaning of the designated skin areas, surface EMG sensors of 16 wireless electrodes (twelve PicoEMG and four Mini Wave Infinity, Wave Plus wireless EMG system, Cometa, Milan, Italy) were positioned on both legs according to the SENIAM guidelines (Seniam.org): vastus lateralis (vast_lat), semitendinosus (sem_tend), gastrocnemius lateralis (gastr_lat), gluteus medius (glut_med), soleus, tibialis anterior (tib_ant), biceps femoris (bic_fem) and rectus femoris (rect_fem).

A 12-camera 3D motion capture system (Vicon, Oxford, UK) was utilized to record marker trajectories with a sampling rate of 200 Hz and EMG data with 1000 Hz simultaneously. Prior to the collection of the static and dynamic trials, participants were required to perform a few tricks in order to become accustomed to skateboarding with attached EMG sensors and markers.

**Figure 1:**
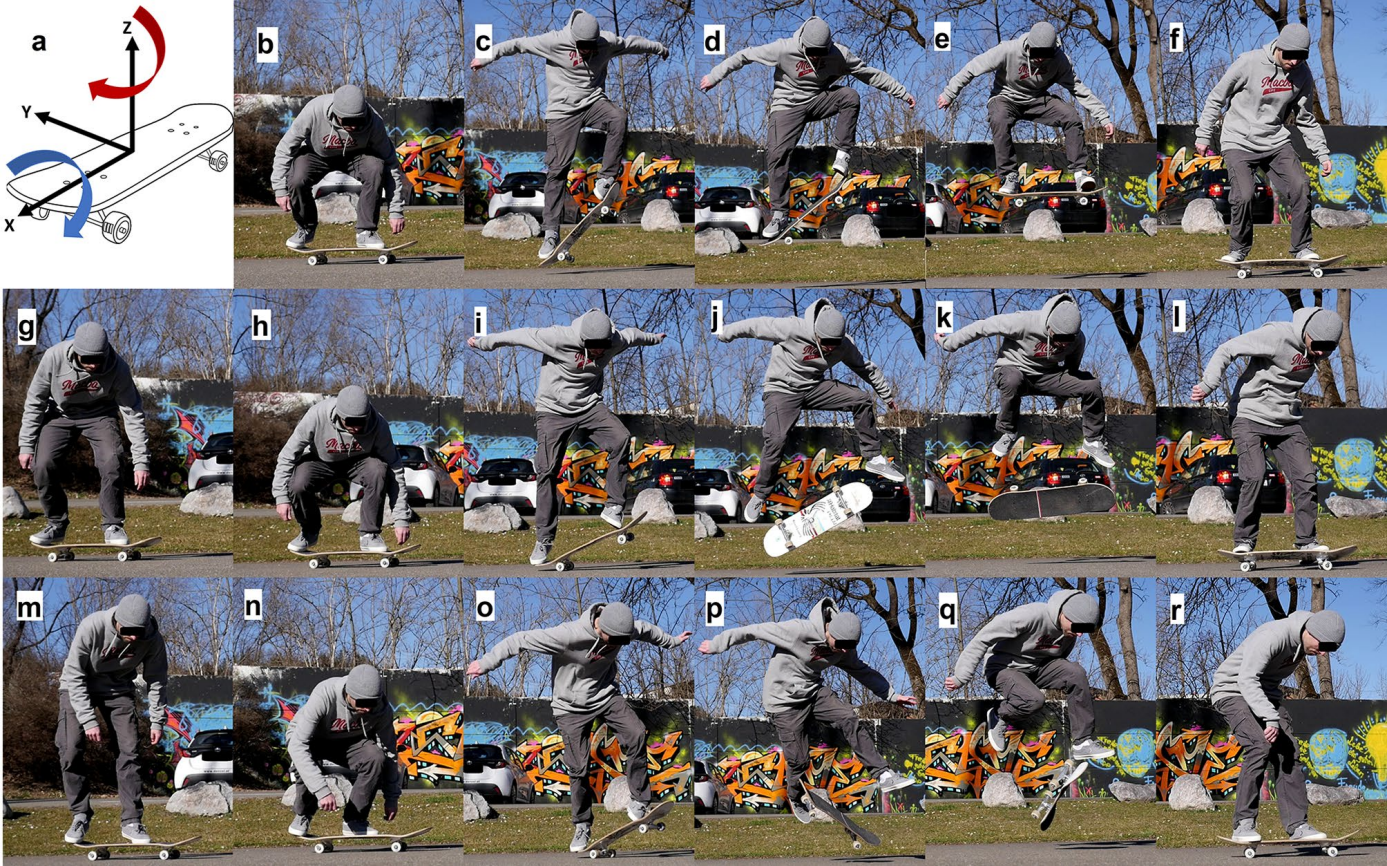
(**a**) The Ollie is a basic jump with the skateboard without any rotation of the board. The board performs a 360° rotation around the x-axis (longitudinal axis) during a Kickflip, and a 360° rotation around the x- axis and the z-axis (vertical axis) during a 360°-flip; (**b**)–(**f**) Ollie; (**g**)–(**l**) Kickflip; (**m**)–(**r**) 360°-flip; the analyzed time frames were between the lowest (**b**, **h**, **n**) and highest (**e**, **k**, **q**) point of the sacrum cluster marker (Supplementary Figure S1). Importantly, this figure shows the tricks performed in a natural environment. Videos of each trick performed in our biomechanics laboratory are provided in the supplementary material of our manuscript.

Each participant performed the following three tricks: an Ollie, a Kickflip and a 360°-flip. These tricks are characterized by their different rotations of the board in the air. An Ollie is a jump without any rotation, whereas a Kickflip involves a 360° rotation around the longitudinal axis of the board, and a 360°-flip involves a 360° rotation around the longitudinal and vertical axes (Fig. 1). These tricks were selected based on their clear progression in difficulty and the common order in which they are typically learned. Among the three tricks, the Ollie serves as the foundation for all others. It typically takes skateboarders several months to perform the Ollie in a proper and consistent manner. The Kickflip generally requires at least 1 year of consistent training, while mastering the 360°-flip typically takes several years of continuous practice. Accordingly, the difficulty of the Ollie, Kickflip, and 360°-flip was assessed as easy, medium, and hard, respectively. Six successful trials per trick were recorded and analyzed. An attempt was considered successful if the participant performed and landed the trick in a clean way, i.e., continued to skate without losing balance. No constraints were imposed on the runout, trick order, speed, jumping height, foot placement on the board, or choice of the front and back leg. This was because skateboarding does not adhere to specific rules regarding the execution of tricks. Participants were permitted as many attempts as they required to successfully perform each trick six times.

### EMG processing

The EMG data was processed using GNU Octave version 6.2.0^32^, similar to the workflow described by Bianco et al.^33^. In detail, the EMG signals were band-pass filtered from 10 to 400 Hz with a 4th-order Butterworth zero lag filter, demeaned, full-wave rectified, and low-pass filtered at 6 Hz with a 4th-order Butterworth zero lag filter. We consider the takeoff phase to be the pivotal period for the success of a trick (Fig. 1 and example videos on the “data availability” link). Consequently, the time interval between the lowest and highest point of the sacrum cluster marker was further analyzed (Supplementary Figure S1). The processed EMG signals were time-normalized to the takeoff phase (100 datapoints) and concatenated, resulting in a m × n matrix for each participant and each trick. Here, m represents the number of muscles (m = 16), while n represents the number of time points (6 trials × 100 points per trial = 600 points). Subsequently, the EMG signals were amplitude-normalized between 0 and 1, with 1 representing the maximum activation amplitude of each muscle across all tricks^21,34^.

### Synergy extraction and determining the number of synergies

For each participant and each trick, muscle synergies were extracted using the “nmf_bpas” octave function^35^. Similar to the classic non-negative matrix factorization^36^, this modified algorithm factorizes the EMG signals into the spatial synergy vectors W, and time-varying activation coefficients C with the smallest possible residual error. The extracted synergy vectors were normalized to a value of 1 based on their maximum values, and the activation coefficients were multiplied by the same normalization values to ensure that their product remained constant^37,38^. As the algorithm can converge at slightly different solutions, depending on the random initialization matrices, the matrix factorization was applied 50 times and the output with the highest tVAF was selected^17,23,39^. Further details on the synergy extraction procedure are provided in the supplementary material.

A total of 1 to 15 (number of muscles − 1) synergies were extracted and NoS was selected according to two criteria: (1) the smallest NoS that ensures a tVAF value exceeding a threshold of 90% and (2) that adding an additional synergy does not increase tVAF by more than 1%. The first criterion is a commonly used approach in human synergy studies^23,37,39–48^. As in previous studies^11,12,23,43,46,48,49^, criterion 2 was employed to address the issue of aiming for considerably higher tVAF values when adding an additional synergy. The NoSoA represents the mean number of synergies that fulfil the threshold criteria for all participants and tricks. The NoSoA was used for all subsequent analyzes (except NoS is specifically referenced) in order to preclude the potential for merging and fractionation, which may arise when extracting different numbers of synergies^50,51^.

### Similarity thresholds

Given the diversity of approaches for quantifying synergy similarities in the literature, we employed three methodologies in this study: cosine similarity CosSim^41,42,51,52^, Pearson’s correlation coefficient r^18,23,39^, and cross-correlation coefficient r_max_^23^. Both W and C were accepted as similar, when the CosSim value of two compared synergies was greater than 0.8^13,49,53,54^. A pair of synergy vectors W was considered to be similar if r > 0.623, which corresponds to the critical value of r for 16 muscles at *p* = 0.01^14–16,18,23,38,51^. A threshold of r_max_ > 0.9 was selected to quantify the similarity between two activation coefficients C, as proposed by Boccia et al.^13^.

### Hypotheses testing

#### NoS and motor complexity of tricks

To ascertain whether the NoS, the tVAF based on NoS, NoSoA and one synergy differed between tricks, repeated-measures ANOVAs were conducted. The Shapiro–Wilk test was employed to verify the normal distribution of the data. If the assumption of normality was not met, the Friedman test was used instead. The Mauchly test was employed to verify sphericity, with the Greenhouse–Geisser correction being applied in the event that sphericity was not given. For pairwise comparisons, the post-hoc Bonferroni correction was employed. The statistical analyses were performed with JASP 0.17.2 (Amsterdam, Netherlands) at a significance level of *p* < 0.05.

#### Inter-participant variability

##### Coefficient of Variation

All inter-participant variability analyses were conducted for each trick separately. To quantify the variability of tVAF and NoS, the coefficient of variation was utilized. It is defined as the ratio between the standard deviation and the mean of the data. Due to its greater sensitivity compared to reporting the standard deviation alone, the coefficient of variation is a widely used metric for assessing inter-participant variability in recent synergy studies^23,41,51,55^. The coefficient of variation was calculated across all participants for NoS and tVAF values based on NoS and NoSoA.

##### Pairwise comparisons

In order to facilitate comparisons between participants, it was necessary to reorder the synergies. The reordering process proposed by Nazifi et al.^12^ was employed to ensure that the most similar synergies were placed in the same order amongst participants (details are provided in the supplementary material). The reordered synergy vectors W and activation coefficients C were then compared between all possible pairs of participants, resulting in 21 W and 21 C comparisons (for seven participants) for each synergy (NoSoA). Subsequently, the values for each synergy were averaged and compared with the established similarity thresholds (section “Similarity thresholds”).

##### Reconstruction of W and C

The W and C of each participant were used to reconstruct the C_rec_ and W_rec_ of all other participants. For instance, the synergy vectors for participant 1 were recomputed by the activation coefficients of all other six participants (resulting in six W_rec_ matrices for participant 1), utilizing the algorithm presented in the supplementary material. Subsequently, tVAF_rec_ was computed using the reconstructed matrix, the corresponding EMG matrix, and the input matrix of the other participant. Next, the degree of similarity between the original W or C matrix and the W_rec_ or C_rec_ matrix was calculated, resulting in six values for each synergy per participant. The values for each synergy (1, …, NoSoA) were then averaged and compared with the pre-established similarity thresholds (section “Similarity thresholds”).

The same procedure was also performed with randomized input matrices. These were preordered according to the six participants, who were required to reconstruct C_rec_ or W_rec_ from each participant. Briefly, randomized matrices were created, reordered based on their CosSim values of corresponding participants, and used to reconstruct the participants’ synergy vectors or activation coefficients (details are provided in the supplementary material). Subsequently, tVAF with randomized inputs (tVAF_rand_) and similarity values across synergies were calculated.

To quantify whether the goodness of tVAF_rec_ can be attributed to similarities between participants or also observed with randomized inputs in a similar manner, a repeated-measures ANOVA was conducted. Thus, the original tVAF of each participant was compared with the averaged tVAF_rec_ of each participant and the averaged tVAF_rand_ of each participant. The assumptions and post-hoc tests were performed in accordance with section “NoS and motor complexity of tricks”.

#### Shared synergies

##### sharedOA—method

In order to examine whether our skateboarders employed similar synergy vectors for different tricks, r was calculated for all possible synergy pairs between two tricks, for each participant. In the sharedOA (shared overall) method, the number of synergies to be compared across tricks was identical for all tricks and participants (NoSoA). Synergies were quantified as shared if the r value was higher than the threshold presented in section “Similarity thresholds” (r > 0.623). Subsequently, the participants’ number of shared synergies was quantified for each trick comparison (n_sharedOA_). The relative number of shared synergies %n_sharedOA_ was calculated as presented in Eq. (1), as proposed by Allen et al.^14–16^. Subscripts trick1 and trick2 indicate the two compared tricks, e.g., Ollie and Kickflip.

1 $$\% n_{sharedOA} = 100 \frac{{n_{sharedOA} }}{{NoSoA_{trick1} + NoSoA_{trick2} - n_{sharedOA} }}$$

##### sharedIND—method

During our analyses, we identified the need for a more individual analysis of shared synergies (sharedIND). To this end, we repeated the sharedOA method with the difference, that NoS was taken instead of NoSoA. This approach has also been used in previous studies^14–16^.

##### Reconstruction of W and C

All reconstruction procedures were conducted separately for each participant. The activation coefficients C_rec_ of each trick were recomputed with synergy vectors W of the other two tricks using the algorithm presented in the supplementary material. To do this, the input matrix W was preordered based on CosSim values of its activation coefficient C and the activation coefficient which should be reconstructed. Similarly, synergy vectors W_rec_ were reconstructed for each trick whit the input matrix C being preordered according to the similarity of its synergy vectors W. This preordering process was necessary as synergies were only ordered among participants per trick, and not across tricks. Following recomputing, similarities between original and reconstructed W or C were computed for each synergy (1, … NoSoA). Subsequently, similarity thresholds (section “Similarity thresholds”) were employed to ascertain whether the synergy was shared or not. Additionally, tVAF_rec_ was calculated to further analyze the reconstruction quality.

The same analyses were performed with randomized input matrices. For example, a randomized synergy vector matrix W_rand_ was created and used to reconstruct the EMG signals of the Kickflip. The reconstructed activation coefficients were then compared with the activation coefficients of the Ollie. Due to CosSim, the matrix W_rand_ was resorted and taken as an input to recompute the activation coefficient C_rec_ of the Ollie. The same method was used in reverse to compute W_rec_ from C_rand_. Furthermore, the degree of similarity between the synergies and the tVAF_rand_ was calculated.

To quantify whether the goodness of tVAF_rec_ can be attributed to similarities between tricks or also appears by chance, a repeated-measures ANOVA was conducted. Thus, the original tVAF was compared with tVAF_rec_ per trick and tVAF_rand_. The assumptions and post-hoc tests were performed in accordance with section “NoS and motor complexity of tricks”.

## Results

**Figure 2:**
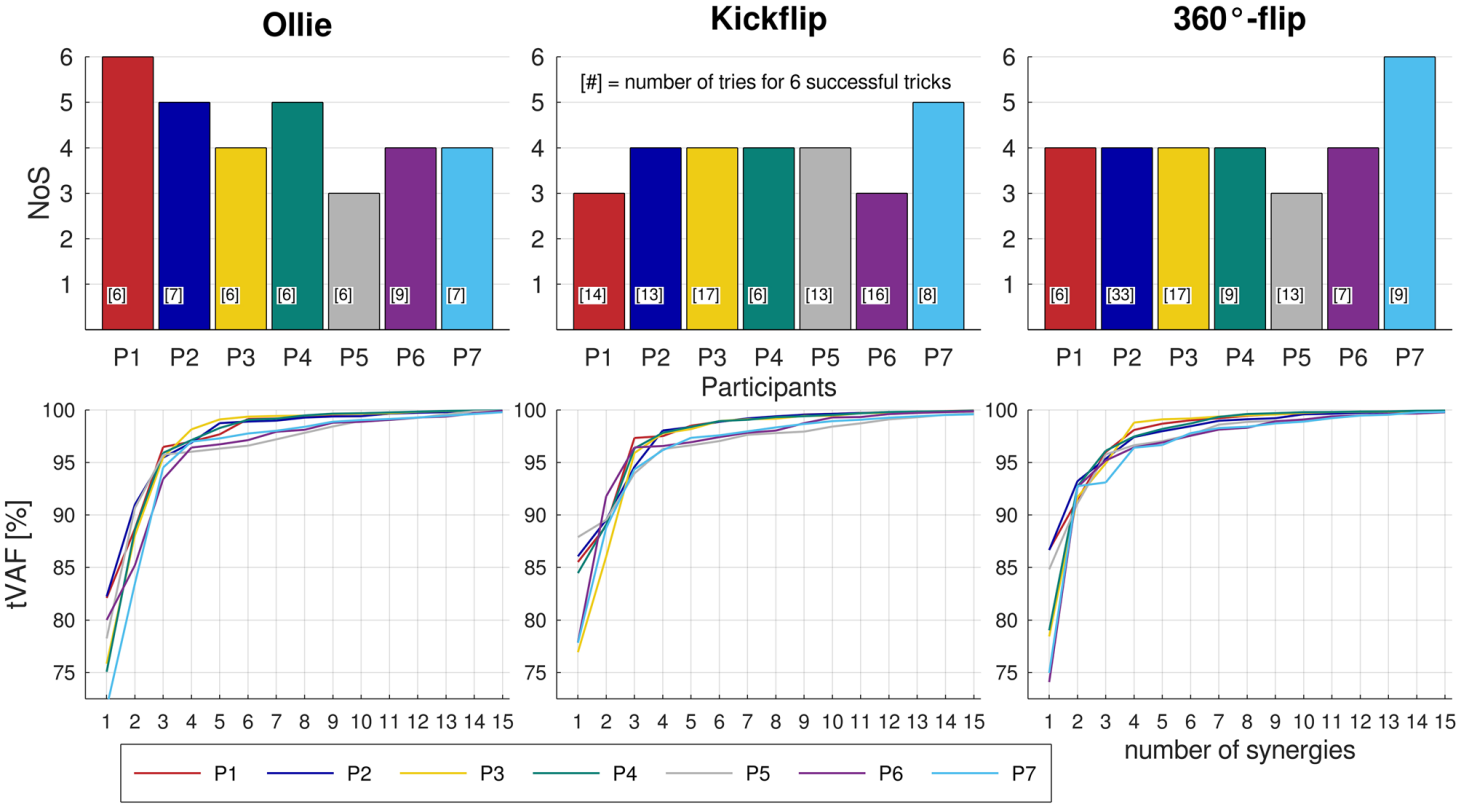
Top row: bars show number of extracted synergies (NoS); [#] show number of attempts for 6 successful trials; Bottom row: total variance accounted for (tVAF) from each participant (P1, …, P7) plotted against number of extracted synergies (1–15).

To perform each trick successfully six times, 6 to 9 (6.71 ± 1.11), 6 to 17 (12.43 ± 4.04), and 6 to 33 (13.43 ± 9.41) attempts were needed for the Ollie, Kickflip and 360°-flip, respectively. The required number of synergies for the tVAF criteria was 3 to 6 (Ollie and 360°-flip) and 3 to 5 (Kickflip) for all participants (Fig. 2). On average, 4 synergies were required, resulting in a NoSoA of 4.

### NoS and motor complexity of tricks

The NoS, the tVAF based on NoS and NoSoA, did not exhibit a significant difference between the Ollie, Kickflip and 360°-flip (Table 1). The ANOVA revealed a significant difference in tVAF at one synergy (*p* = 0.025). The values were smaller for the Ollie (77.88 ± 3.92) compared to the Kickflip (82.37 ± 4.64; *p* = 0.024), but no significant difference was observed between the 360°-flip (80.67 ± 5.37) and any other trick.

**Table 1:** Coefficient of variation (CoV), mean (M) and standard deviation (SD) of NoS, tVAF of NoS and NoSoA.

Trick	NoS			tVAF NoS			tVAF NoSoA		
	CoV	M	SD	CoV	M	SD	CoV	M	SD
Ollie	0.22	4.43	0.98	0.01	97.64	1.27	0.01	96.94	0.66
Kickflip	0.18	3.86	0.7	0.01	97.3	0.69	0.01	97.17	0.8
360°-flip	0.22	4.14	0.9	0.01	97.4	1.02	0.01	97.32	0.9

### Inter-participant variability

**Figure 3:**
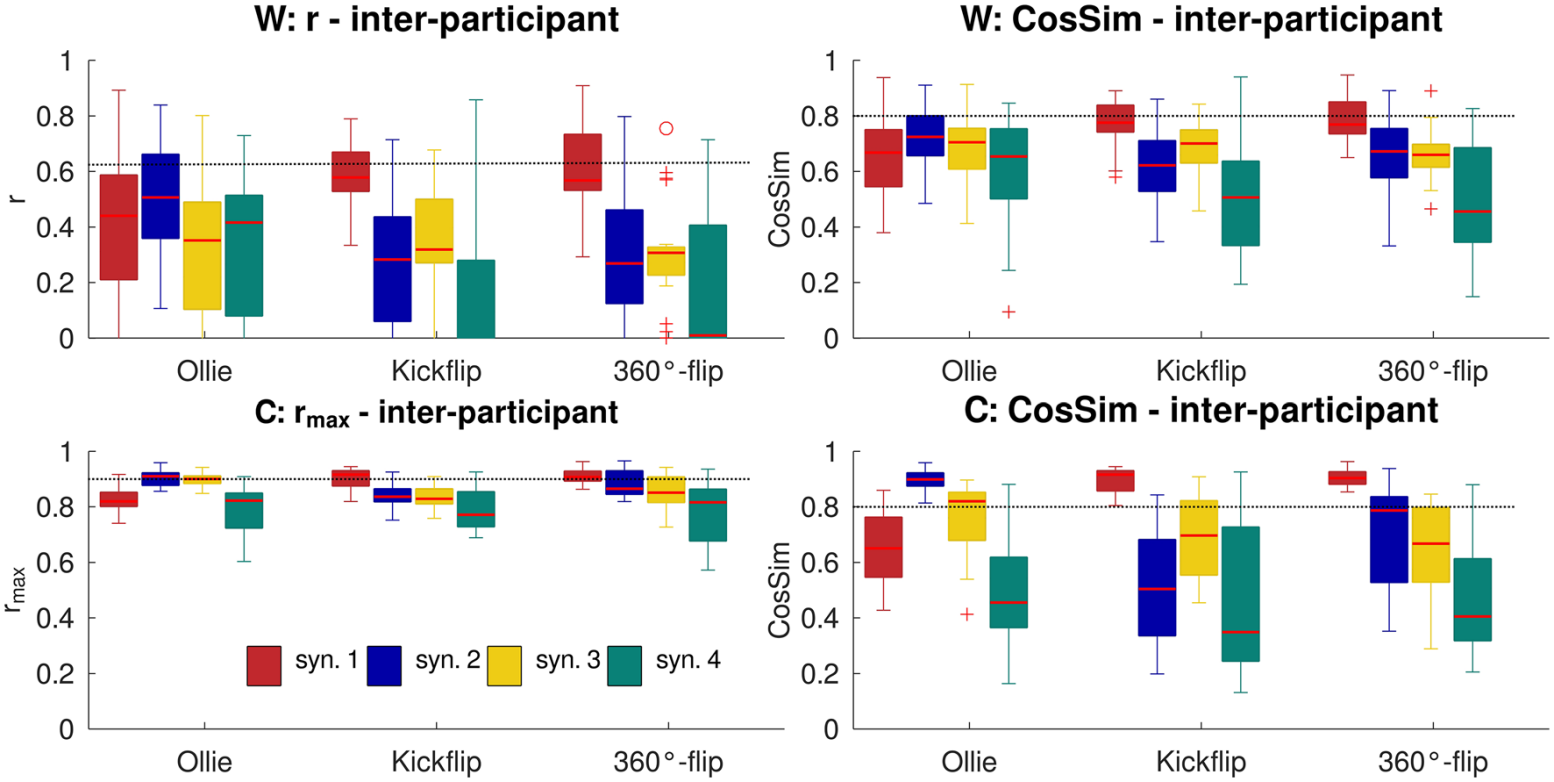
Boxplots showing the variability in synergy vectors (W, top row) and activation coefficients (C, bottom row) between participants for synergies (syn.) 1 to 4. Horizontal lines indicate chosen similarity thresholds: Pearson correlation coefficient (r) > 0.623, cross-correlation coefficient (r_max_) > 0.9, cosine similarity (CosSim) > 0.8.

The coefficient of variation values ranged from 0.18 to 0.22 for NoS, and were 0.01 for the tVAF based on NoS and NoSoA (Table 1). Across all tricks, the r, r_max_ and CosSim values, were above our similarity thresholds for only three activation coefficients and none of the synergy vectors (Fig. 3). In detail, C was similar for synergy #2 of Ollie (CosSim = 0.9 ± 0.04; r_max_ = 0.91 ± 0.03), synergy #1 of Kickflip (CosSim = 0.89 ± 0.05; r_max_ = 0.9 ± 0.05), and synergy #1 of 360°-flip (CosSim = 0.91 ± 0.03; r_max_ = 0.91 ± 0.03). The robustness of W was tested by comparing the original C with C_rec_. For each trick, the similarity of the matrices exceeded the pre-established thresholds for the following synergies (# indicates the synergy number; Supplementary Figure S2):

- Ollie: **#2**: CosSim = 0.89 ± 0.08; r_max_ = 0.92 ± 0.05; **#3**: CosSim = 0.87 ± 0.08; r_max_ = 0.91 ± 0.06
- Kickflip: **#1**: CosSim = 0.93 ± 0.07; r_max_ = 0.95 ± 0.03; **#3**: CosSim = 0.8 ± 0.12
- 360°-flip: **#1**: CosSim = 0.91 ± 0.07; r_max_ = 0.93 ± 0.04; **#3**: CosSim = 0.8 ± 0.13

The robustness of C was tested by comparing the original W with W_rec_. For each trick, the similarity of these matrices exceeded the thresholds for the following synergies (Supplementary Figure S2):

- Ollie: **#2**: CosSim = 0.95 ± 0.05; r = 0.91 ± 0.1; **#3**: CosSim = 0.86 ± 0.16; r = 0.72 ± 0.3
- Kickflip: **#1**: CosSim = 0.96 ± 0.03; r = 0.93 ± 0.07
- 360°-flip: **#1**: CosSim = 0.97 ± 0.02; r = 0.95 ± 0.03; **#2**: CosSim = 0.81 ± 0.21

**Figure 4:**
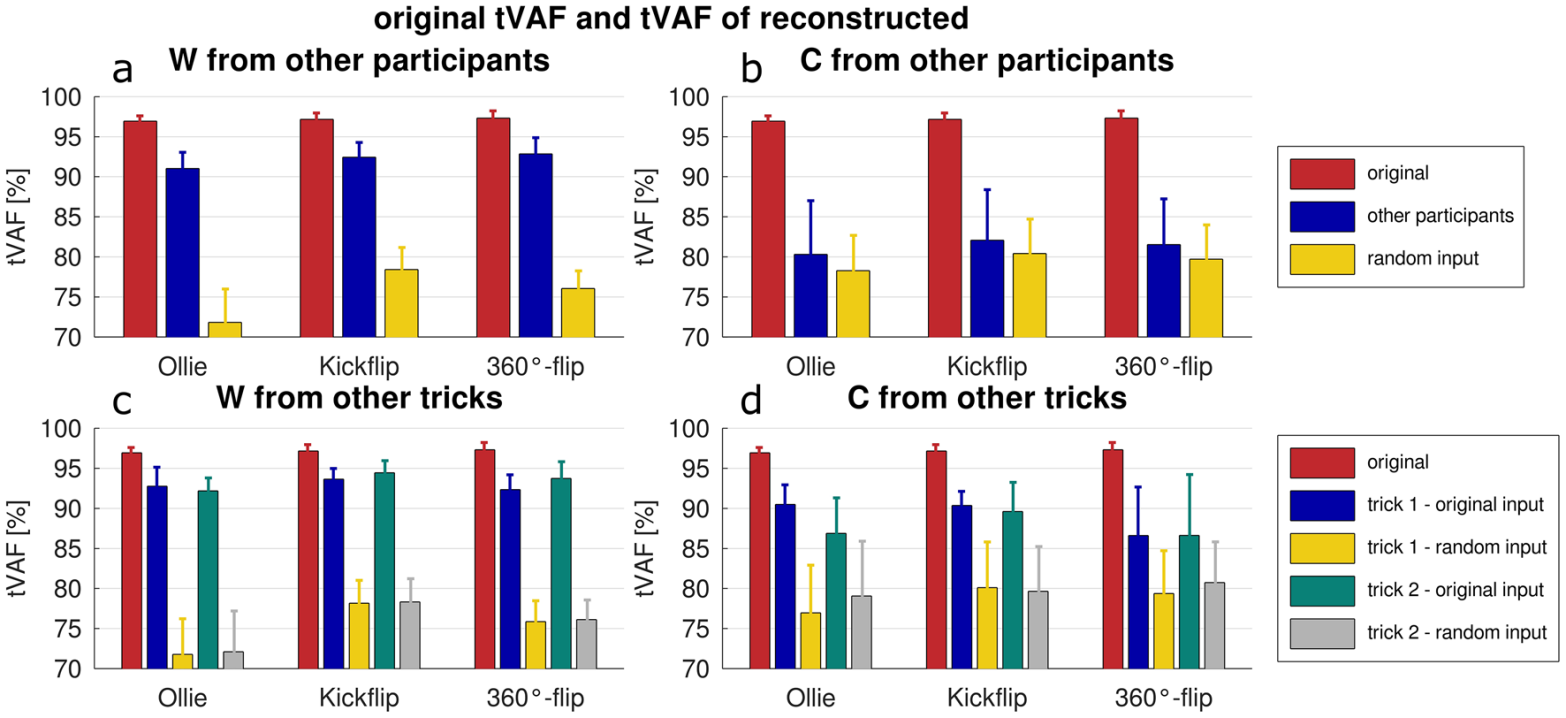
Mean (bars) and standard deviation (error bars) of the original and reconstructed total variance accounted for (tVAF). Top subplots show original tVAF and tVAF of reconstructed synergy vectors (W, subplot a) or activation coefficients (C subplot b) from other participants or random inputs. Bottom subplots: original tVAF and tVAF of reconstructed W (subplot c) or C (subplot d) from other tricks (trick 1 and trick 2).

When activation coefficients were reconstructed from random values, C_rec_ and W_rec_ were not similar to the original matrices in any case. The repeated-measures ANOVA comparing tVAF with tVAF_rec_ and tVAF_rand_ showed significant differences (*p* < 0.001–0.002) for all comparisons: Ollie W_rec_ & C_rec_, Kickflip W_rec_ & C_rec_, 360°-flip W_rec_ & C_rec_. Pairwise comparisons revealed that, when the reconstructions of W were performed using C from other participants, the original tVAF exhibited the highest value, followed by tVAF_rec_, with tVAF_rand_ matrices showing the lowest values (*p* < 0.001–0.006; Fig. 4). When C was reconstructed by W from the other participants—for Ollie and Kickflip—tVAF was higher than tVAF_rec_ and tVAF_rand_ (*p* < 0.001), with no significant difference between tVAF_rec_ and tVAF_rand_. For 360°-flip, tVAF was only higher than tVAF_rand_ (*p* < 0.014; Fig. 4).

### Shared synergies

#### sharedOA—method

**Figure 5:**
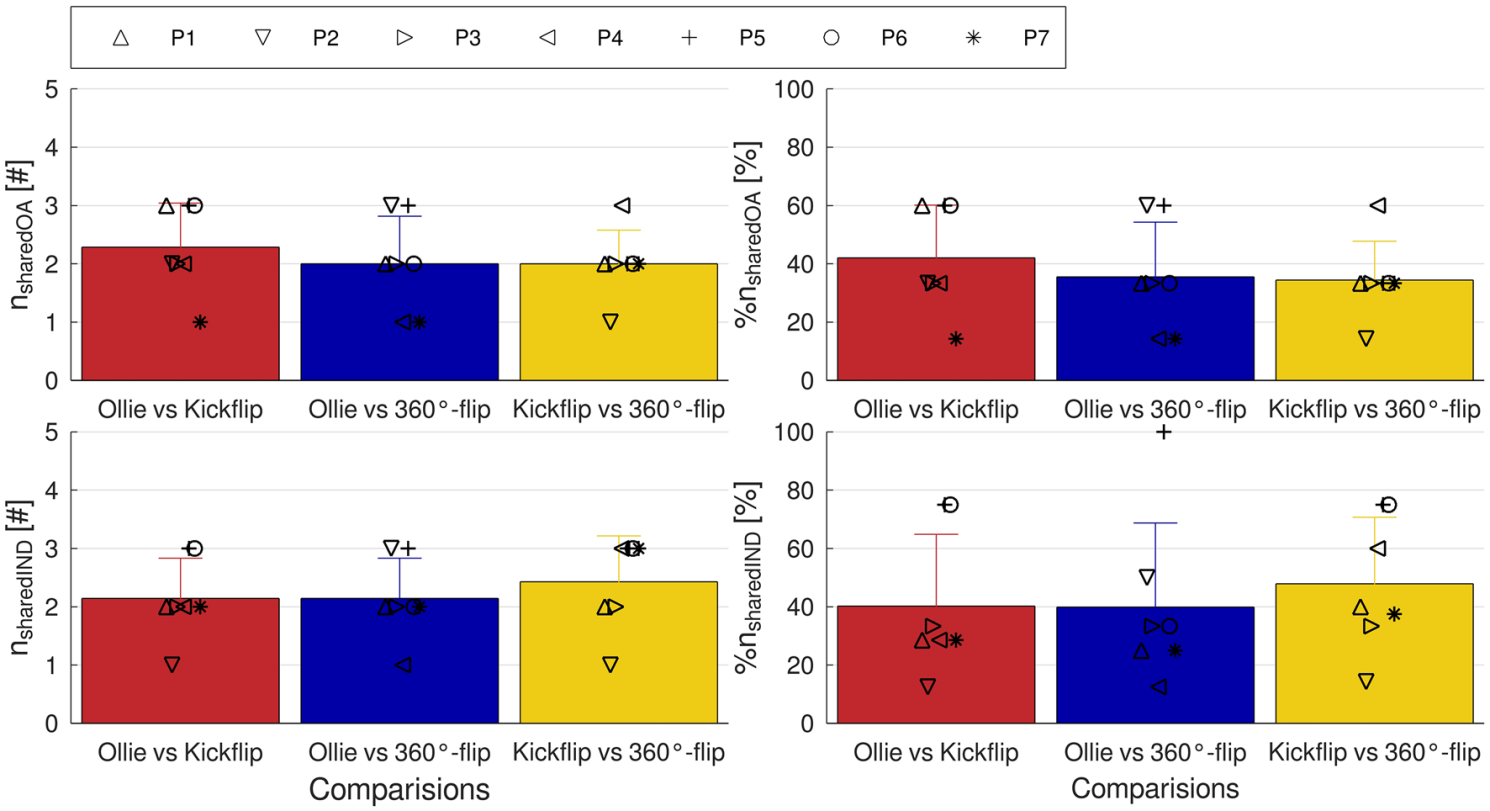
Absolute [#] and relative [%] number (n) of shared synergies between tricks; means across participants are plotted as bars, with standard deviation as error bars; markers indicate individual values for participants (P1, …, P7). Top row: results based on the sharedOA (shared overall) method. Bottom row: results based on the sharedIND (shared individual) method.

A high variability was observed in the amount of shared synergies, both between participants and between trick comparisons (Fig. 5). The number of shared synergy vectors W ranged from 1 to 3 for all comparisons between tricks. In no instance were all synergies shared or none shared.

#### sharedIND—method

As with the sharedOA—method, the sharedIND—method demonstrated considerable variability between participants and trick comparisons (Fig. 5). The range of shared synergies was between 1 and 3 for all trick comparisons. All synergy vectors were shared by only one participant for Ollie vs 360°-flip, while no participant shared none of the synergy vectors in any comparison.

#### Reconstruction of W and C

According to the pre-established similarity threshold for CosSim (> 0.8), the reconstructed matrices of another trick were found to be similar to the original matrices for 2 to 4 synergies for C_rec_ and 1 to 4 synergies for W_rec_. When applying the r_max_ > 0.9 and r > 0.623 thresholds, the number of similar synergies was found to be generally lower, with a range of 1 to 4 synergies. Once more, the variability between participants and trick comparisons was considerably high (Supplementary Table S1). With randomized preordered input matrices, the similarity for C_rec_ and W_rec_ ranged from 0 to 4 synergies according to CosSim. With regard to r_max_ and r thresholds, the number of similar synergies was in general lower, and ranged from 0 to 3 and 0 to 4 for C_rec_ and W_rec_, respectively. The number of similar synergies with randomized inputs was, in the majority of cases, lower than the number of similar synergies with original inputs (Supplementary Table S1).

The comparisons of tVAF with tVAF_rec_ by the other two tricks and with tVAF_rand,_ were statistically significant (*p* < 0.001) for all tests: Ollie W_rec_ & C_rec_, Kickflip W_rec_ & C_rec_, 360°-flip W_rec_ & C_rec_. Pairwise comparisons revealed that tVAF was always significantly higher than tVAF_rand_ (*p* < 0.001–* p* = 0.01) and tVAF_rec_ was significantly higher than tVAF_rand_ (*p* < 0.001–0.044), with the exception of the 360°-flip W_rec_ & C_rec_. In the case of Ollie W_rec_ & C_rec_, there was no significant difference between tVAF and tVAF_rec_ when matrices were reconstructed by the Kickflip. For the Kickflip W_rec_ tVAF and tVAF_rec_ were not significantly different when matrices were reconstructed by the 360°-flip. Furthermore, tVAF and tVAF_rec_ were not significantly different for the 360°-flip W_rec_ and Kickflip C_rec_. In all other cases, tVAF was found to be significantly higher than tVAF_rec_ (*p* = 0.001–0.031). There was no significant difference between the two tVAF_rec_ values, nor between the two tVAF_rand_ values created by the reconstruction of one trick by the other two (Fig. 4).

## Discussion

The objective of this study was to gain further insights into the neuromuscular coordination of movements, with a high level of complexity, specifically three different skateboard tricks. Our results showed that (i) the number of required synergies remained unchanged across tricks, (ii) the synergies were highly variable between participants, and (iii) the synergies were shared across tricks in each participant.

### NoS and motor complexity of tricks

Contrary to our initial hypothesis, the NoS and the tVAF at NoS and at NoSoA did not differ between the three tricks. One possible explanation for this unexpected finding is that, following a period of learning, the motor complexity of these tricks is equivalent.

It is assumed that the number of required synergies increases with an increasing level of complexity. Several studies have demonstrated that populations with impairments, such as stroke survivors or children with cerebral palsy, use fewer synergies to perform movements compared to typically developing individuals^8,9,56,57^. Furthermore, the NoS increased as the degree of impairment decreased^58^. In a study by Dominici et al.^59^, it was found that only two synergies were sufficient for neonates stepping, while four synergies were needed to explain gait patterns of toddlers and adults. When these findings are considered in the context of our study, it can be concluded that there is no difference in the motor complexity of the tricks. One potential explanation for the similar motor complexity observed across different tricks is that an additional synergy is not added to the existing synergies, but rather that other synergies are used. For instance, instead of adding an extra subtask to the Ollie to rotate the board in the air, which is the case for the Kickflip and 360°-flip, some subtasks are modified. As subtasks are learned, their muscle activation blocks are stored in the spinal cord^1–3^ as quasi-fixed synergy vectors, which can be activated by central stimuli. Consequently, the motor complexity does not necessarily has to increase when rotations of the board are performed in comparison to an Ollie. This theory is supported by our results, which demonstrated that only a few synergies were shared between tricks and that each trick included several task-specific synergies.

The analysis of tVAF at one synergy challenges our proposed conclusion of similar motor complexity across tricks. Previous research has demonstrated that cerebral palsy patients exhibit higher tVAF at one synergy during walking, suggesting a lower motor complexity compared to unimpaired individuals^8,60^. In the present study, the Ollie exhibited a lower tVAF at one synergy compared to the Kickflip. This suggests that the Ollie, the trick that we predefined as the easiest based on its learning time, exhibits a higher motor complexity. Consequently, we called into question our proposed ranking on trick difficulty and employed a different approach, defining the tricks’ difficulty based on the number of required attempts to perform them successfully six times. This subsequent analysis, presented in the supplementary material, revealed significant differences in the ANOVA, but not in the post-hoc pairwise comparisons. The mean and individual number of attempts (Fig. 2) indicates that the Ollie is the trick with the lowest difficulty, while the Kickflip and the 360°-flip exhibited considerable inter-individual differences in difficulty. Consequently, we are confident that the Ollie serves as the easiest and basic trick, while the other two show participant-specific difficulties. In light of these findings and the absence of differences in NoS and tVAF at NoS among tricks, we hypothesize that tVAF at one synergy solely reflects the level of muscle coactivation, which may not necessarily indicate differences in motor complexity. Furthermore, we maintain that motor complexity is similar across tricks.

### Inter-participant variability

A high inter-participant variability in synergies was observed. The coefficient of variation values for all tVAF measures were relatively low (1%) and comparable to or even lower than those reported in previous studies^41,51,55^. In contrast, the coefficient of variation values for NoS were higher than for tVAF, which is in agreement with the literature. Previous studies have reported coefficient of variation values of approximately 15% for hand grasps^41^, up to 12.6% for walking^55^, and around 20% for upper limb reaching movements, which was decreased to 15% when outliers were excluded^51^. In our study, we observed rather high coefficient of variation values for NoS, with values of 22%, 18% and 22% for the Ollie, Kickflip and 360°-flip, respectively (Table 1).

Across all three tricks and all participants, no synergy vector and only three activation coefficients met the established similarity criteria. This high inter-participant variability is likely due to two key factors: (1) the chosen methodology for defining inter-participant variability, and (2) the absence of constraints for movement execution.

(1) The used workflow may influence the inter-participant variability outcomes. Prior to the comparison of synergies, matrices were preordered to one reference participant. While analogous approaches were employed by other researchers^23,41,51^, we assume that higher similarities across participants would be observed if synergies were not compared after an ordering procedure, but by pairs of two. A closer examination of the values of the pairwise comparisons revealed that the synergy vectors were quite similar between some participants, while they were rather different among others. Furthermore, the boxplots in Fig. 3 demonstrate that a considerable number of values exceeded the selected thresholds. However, the high range of the data resulted in a reduction of the mean and the median, which fell below the thresholds. Consequently, it can be hypothesized that some participants employed motor strategies that were more similar than the results initially suggest. Another factor that may influence the findings is the selection of threshold values. While threshold values are well defined and accepted for r (see section “Similarity thresholds”), general cut-off values for CosSim do not yet exist. Here, a threshold of > 0.8 was chosen in line with other researchers^13,53,54^. Nevertheless, other thresholds have been employed in previous studies, including > 0.75^17,50^ and > 0.7^12^. When comparing our CosSim results with our r and r_max_ values, the selected threshold appears to be appropriate. However, it is important to consider the potential impact of differing thresholds when comparing our findings with those of other studies.

(2) The movement execution of our skateboarders was not constrained. Previous studies have found high inter-participant similarities for cycling^39^ and hand grasps^41^. Even in complex movements, which require years of training, high similarities were found. In detail, Frère & Hug^23^ found high consistency for two out of three synergies between nine gymnasts while they performed backward giant swings. All these studies included constrained movements. In contrast to the aforementioned studies, barely any constraints were imposed upon the skateboarders in our study, which may have contributed to the observed low inter-participant similarity values. Our findings align with those of Zhao et al.^51^ and Scano et al.^61^, who also reported low inter-participant similarity in minimally constrained upper limb movements. This is consistent with one of Bernstein’s concepts of movement construction, namely that movement goals can be achieved through different motor strategies when there are few constraints^62^.

While no similar synergy vectors were observed, three activation coefficients were found to be consistent across participants. Additionally, the tVAF results were contingent upon the input matrices. When reconstructing synergy vectors by activation coefficients, tVAF_rec_ exceeded VAF_rand_. However, tVAF_rec_ and VAF_rand_ values were not significantly different when activation coefficients were reconstructed by synergy vectors. Consequently, the observed similarities of synergy vectors could also be attributed to chance. The higher consistency of activation coefficients compared to the lower similarity of synergy vectors suggests that the timing, is more consistent across participants compared to the actual synergy vector. One potential explanation is that synergy vectors, such as those required to rotate the board, must be activated during the same time span. In contrast, the actual muscle weightings within synergies may differ due to differences in movement techniques.

### Shared synergies

**Figure 6:**
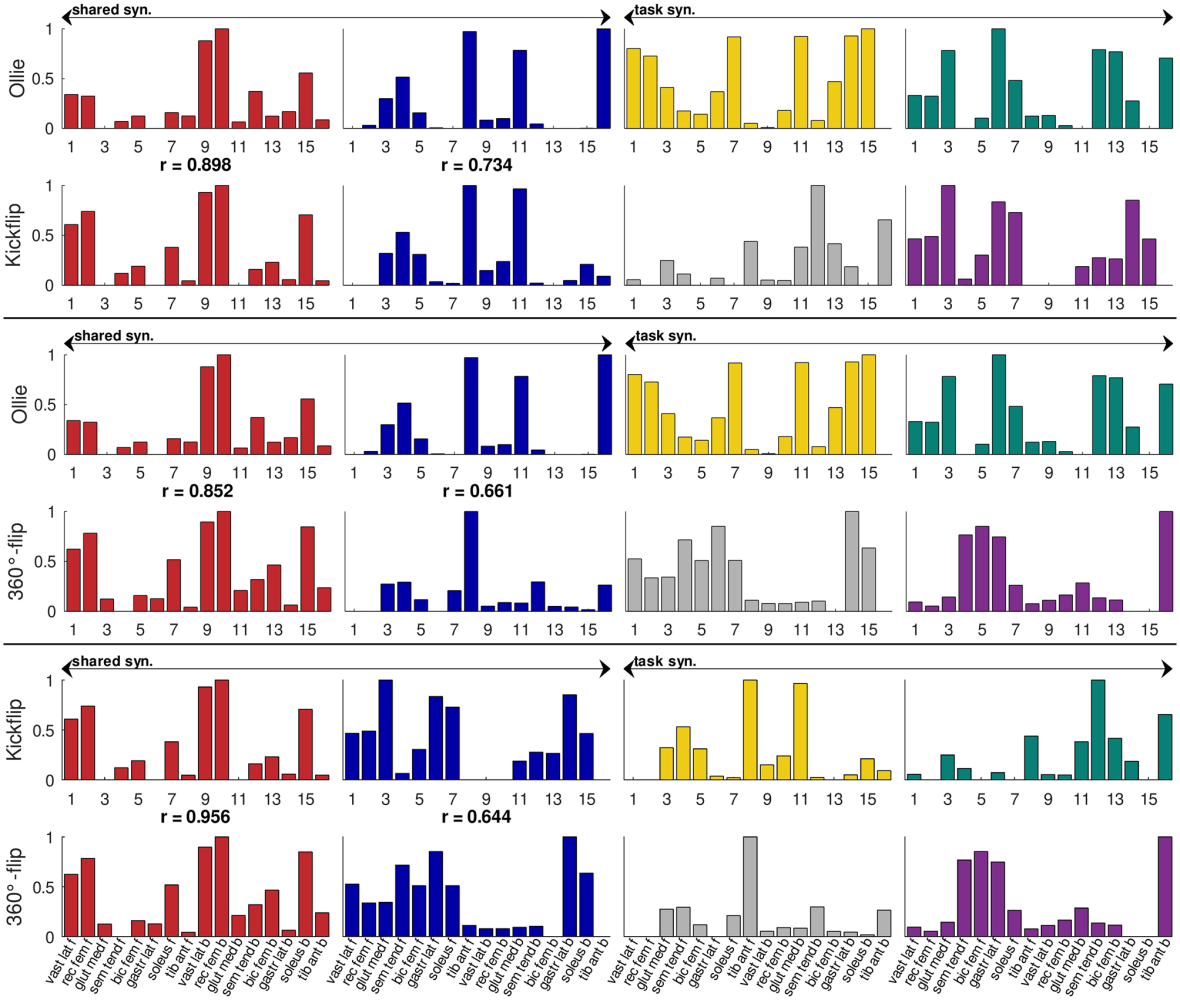
Shared and task-specific synergies (syn.) quantified with the sharedOA method between Ollie and Kickflip, Ollie and 360°-flip, Kickflip and 360°-flip for one participant; r = Pearson’s correlation coefficient; y-ticks indicate the muscle weight, x-ticks indicate the muscles for the front (f, muscle 1–8) and back (b, muscle 9–16) leg; red syn. is consistent across all tricks.

**Figure 7:**
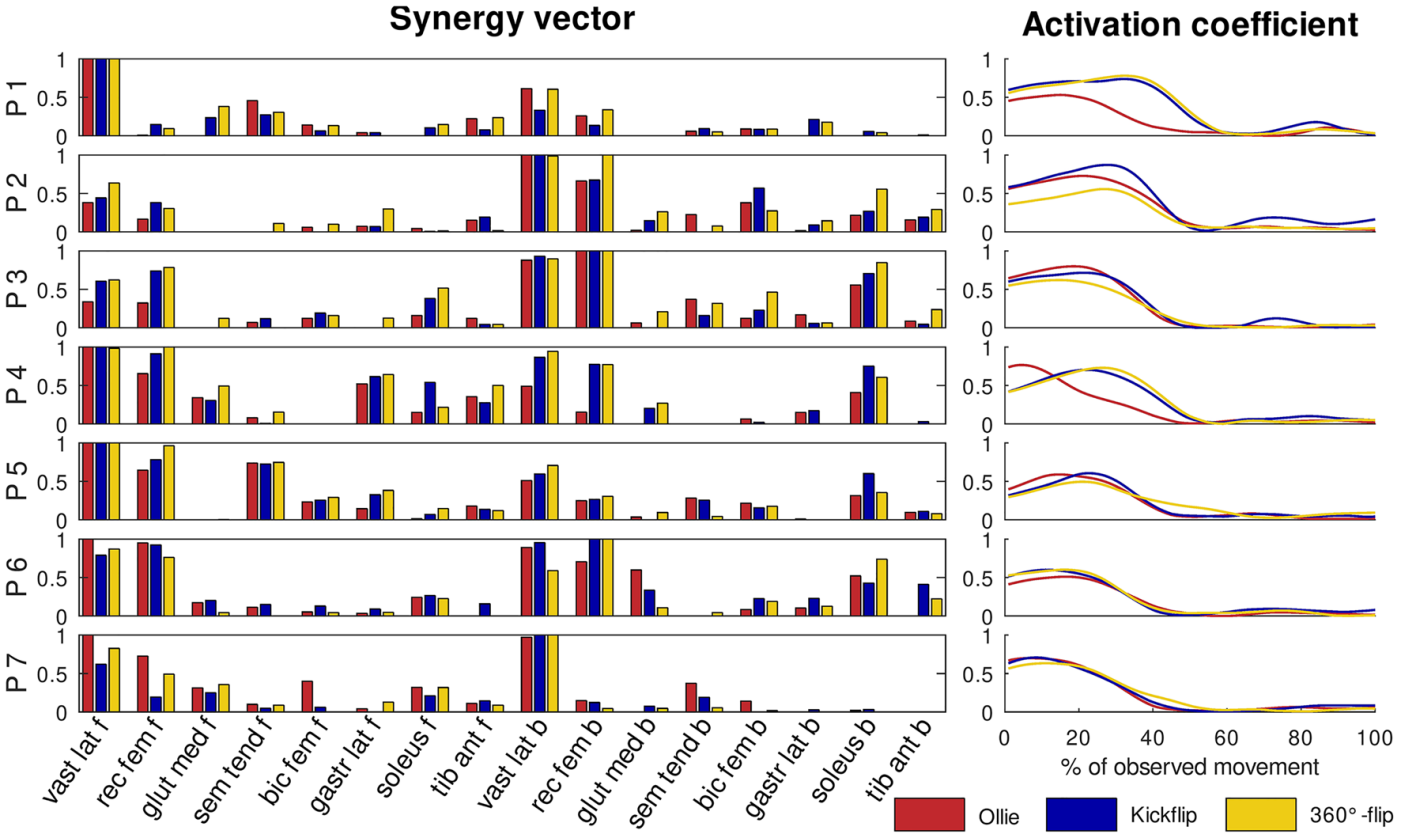
Synergy vectors and activation coefficients which were shared across all three tricks of all participants (P1 … P7) based on the sharedIND method; y-ticks (0–1) are the muscle weightings (left subplots) or level of activation (right subplots) for synergy vectors or activation coefficients. Each waveform indicates the average activation coefficient across trials per trick. The time interval between the lowest (0%) and highest (100%) point of the sacrum cluster marker was analyzed (% of observed movement, see supplementary Figures S1). f = front leg; b = back leg.

Regardless of the method used to quantify similarities (sharedOA or sharedIND), shared synergies between tricks were present. Figure 6 illustrates shared and task-specific synergy vectors of participant 3 (sharedOA) and shows that one synergy (in red) is consistent across all tricks. At least one synergy vector was also shared between all three tricks in all other participants, regardless of the method used. Upon closer examination, it was observed that the consistent synergy vector across all three tricks was primarily activated in the first third of the movement and emerged from the vastus lateralis and rectus femoris muscles from both the front and back legs (Fig. 7, supplementary Figure S3). In light of these findings, we propose that the shared synergies, which were observed across all three tricks, were responsible for the ‘basic movement’ of the jump to elevate the board. The task-specific synergies, which were primarily composed of other muscles (Fig. 6, Supplementary Figure S4), exhibited their highest activation levels after the ‘jump’ synergy. Consequently, task-specific synergies present modulated muscle coordination to rotate the board. While the precise adaptations occurring during learning processes remain unclear, we assume that during the acquisition of a new skill, fundamental synergies remain unchanged, while only some synergies responsible for movement fine-tuning are newly formed. Nevertheless, further research in the field of motor learning is required to substantiate this hypothesis, which is based solely on the case of skateboarding.

The results of the reconstruction, namely W_rec_ and C_rec_, confirm our findings, that shared synergies exist across tricks. Firstly, a comparison of the reconstructed matrices with the original matrices revealed similarities for every condition (Supplementary Table S1). Secondly, tVAF_rec_ values were not always significantly different from the original tVAF (Fig. 4). Surprisingly, analyses based on random inputs demonstrated that similarities could also occur by chance. Furthermore, some tVAF_rand_ values were found to be similar to tVAF_rec_ values. Additionally, comparisons of the original synergy vectors and activation coefficients with their reconstructed matrices by random inputs frequently exceeded the established similarity thresholds (Supplementary Table S1). These ‘similarities by chance’ were observed more frequently for CosSim than r and r_max_. Consequently, we propose that r and r_max_ should be employed to identify similarities between synergy vectors and activation matrices, or that the CosSim thresholds should be modified.

In summary, we have identified shared synergies between different skateboarding tricks, which require years of specific training. Our findings align with previous research indicating the presence of similar synergies among movements with similar subtasks^1,12^. However, to the best of our knowledge, this is the first study to demonstrate that shared synergies are utilized in complex and minimally constrained motor tasks.

### Limitations

Our study included the following limitations. Firstly, determining the required NoS is a challenging task. While tVAF is commonly used for this purpose, its primary limitation lies in the typically arbitrary selected threshold^63^. Upon closer examination of the tVAF curves (Fig. 2), we observed that, in contrast to a smooth increase in tVAF with additional synergies, there were instances of significant jumps (e.g., 360°-flip of P7). These sharp jumps are attributed to the splitting of a particular synergy when salient features are taken into account^64^. To ensure the inclusion of synergies capturing important features following such sharp jumps in the tVAF curve, we applied our second criterion to determine an appropriate NoS. Namely, that adding an additional synergy does not increase tVAF by more than 1%. Although these thresholds were arbitrarily chosen, similar to previous studies^11,23,49^, we believe our analyses encompassed the most significant synergies for the performed movements. Secondly, our study included only seven participants. This sample size is within the typical range of five to twelve participants in studies focusing on inter-participant variability analyses of muscle synergies^11,23,39,51,55,65^. While we assume that our results are unlikely to undergo drastic changes with a larger cohort of participants, it is essential to consider the small number of participants when interpreting the findings of our study. Thirdly, the participants performed the tricks on a wooden track in the biomechanics laboratory, which differed from their natural skateboarding environment in terms of space and friction coefficients. Skateboarders frequently skate on a variety of surfaces with varying friction coefficients, including concrete, indoor skateparks with wooden floors and obstacles, and other similar environments. Furthermore, the analyses were conducted to compare the tricks performed under identical experimental conditions. Consequently, we are confident that the laboratory setting did not introduce a bias into our findings or main conclusions. Fourthly, it should be noted that the analyses focused exclusively on skateboarding tricks, which were selected as representative tasks for highly complex movements. Given the highly specific nature of skateboarding tricks and the limited number of participants, further research on other complex movements and with a larger cohort is encouraged to confirm the conclusions drawn here.

## Conclusion

Based on the insights gained from our study, we draw the following conclusions: (i) The motor complexity remained unchanged across tricks. (ii) The inter-participant variability is high in movements with few constraints due to the existence of numerous possible solutions to achieve the same goal. (iii) Shared and task-specific synergies exist in different skateboard tricks. Notable is the consistency of one shared synergy vector across all three tricks in each participant, which might be responsible for a fundamental subtask like jumping. The findings of this study offer novel insights into the motor control of highly complex movements, providing a foundation for further research, particularly in the field of motor learning.

### Supplementary Information

Supplementary Information 1.

Supplementary Information 2.

## Data Availability

The anonymized EMG input data, i.e. raw and processed EMG signals and the events (start and end of analyzed movements) are available on https://osf.io/ybv48/?view_only=0d4c500aeebc4466a0e379df0f8e0a1a. For c3d files and Octave codes contact Paul Kaufmann at paul.kaufmann@univie.ac.at or Hans Kainz at hans.kainz@univie.ac.at. The authors will assist with any reasonable replication attempts for 2 years following publication.
