# Supplementary material for: Muscle synergies are shared across fundamental subtasks in complex movements of skateboarding
Source: Sci Rep. 2024 Jun 4;14:12860. doi: 10.1038/s41598-024-63640-5 (PMC11150461; doi:10.1038/s41598-024-63640-5)
Supplement: Supplementary file 1 — Supplementary Information 1. [file 41598_2024_63640_MOESM1_ESM.pdf]

Supplementary Material

Muscle synergies are shared across fundamental subtasks in complex movements of skateboarding

Paul Kaufmann[1,2], Lorenz Zweier[1,2], Arnold Baca[1], Hans Kainz[1,2*]

[1]Department of Biomechanics, Kinesiology and Computer Science in Sport, Centre for Sport Science and University Sports, University of Vienna, Vienna, Austria

[2]Neuromechanics Research Group, University of Vienna, Vienna, Austria

[*] Corresponding author:
Ass.-Prof. Mag. Hans Kainz, MSc PhD

hans.kainz@univie.ac.at

Head of the Neuromechanics Research Group, University of Vienna

https://neuromechanics.univie.ac.at/

University of Vienna, Department of Biomechanics, Kinesiology and Computer Science in Sport, Centre for Sport Science and University Sports

Auf der Schmelz 6a (USZ II), 1150 Vienna, Austria

1 Synergy extraction: non-negative matrix factorization

Slightly different algorithms of non-negative matrix factorization [1-3] are commonly used and accepted for extracting muscle synergies from EMG matrices [4-9]. The objective of the algorithm is to model muscle activity ($E_{m\times n}$) as a linear combination of synergy vectors ($W_{m\times k}$) and activation coefficients ($C_{k\times n}$) with a minimal residual error (e), as shown in equation (1). Note that k represents the number of extracted synergies and ranges from 1 to 15 (number of muscles -1) in the current study, while g represents the synergy number.

$$(1) \quad E_{16\times 600} = \sum_{g=1}^{k} C(g)_{k\times 600}\, W(g)_{16\times k} + e$$

In our study, we employed an advanced non-negative matrix factorization algorithm, introduced by Kim & Park [10], based on the block principal pivoting method for the non-negativity constrained least squares problem. In contrast to the classic algorithm [2], which finds a local minimum, convergence is reached as a stop criterion. Hence, we slightly modified the “nmf_bpas” octave function [10] for our purposes. In detail, 50 to 5000 iterations were permitted, to reach a convergence criterion of 10^{-6} for f in equation (2). Subscript F indicates the Frobenius norm and α represents the mean of E. As the algorithm can converge at slightly different solutions, depending on the random initialization matrices, we applied the matrix factorization 50 times and selected the run with the highest tVAF [5, 11, 12]. The extracted synergy vectors were normalized to a value of 1 based on their maximum values, and the activation coefficients were multiplied by the same normalization values to ensure that their product remained constant [13, 14].

$$(2) \quad f(W,C) = \frac{1}{2}\left(\|E - WC\|_F^2 + \alpha\,\|W\|_F^2 + \alpha\|C\|_F^2\right)$$

2 Synergy ordering

In order to facilitate comparison between W and C across participants, it was necessary to reorder the synergies. The reordering process proposed by Nazifi et al. [9] was employed to ensure that the most similar synergies were placed in the same order for each participant. In detail, CosSim was calculated for all possible synergy pairs between two participants. The highest CosSim values were then identified, and the corresponding synergies were sequentially removed until none remained. In the subsequent step, a reference participant was selected based on the greatest number of CosSim values exceeding 0.7. The reference participants’ synergies were reordered with the highest number of CosSim values exceeding 0.7 at the top and the lowest number at the bottom. Finally, the synergies of the other participants were reordered based on their CosSim pairs with the reference participant.

3 Recomputing of synergy vectors and activation coefficients

To assess the robustness of W and C across participants per trick and across tricks per participant, a reconstruction algorithm for W and C was employed, which is commonly used in the literature [12, 15-19]. This algorithm is based on the updating rule from Lee & Seung [3] for non-negative matrix factorization. As shown in Equations (3) and (4), the C or W from one condition was held fixed (suffix: fix) to reconstruct W or C (suffix: rec), respectively, with E from another condition. Following an initial random guess for the reconstruction matrix, numerous iterations (n) were conducted until the function f(W,C) reached a convergence criterion. Subscripts i and j indicate the row and column, while

superscript T indicates the transposed matrix. The number of iterations permitted ranged from 50 to 1000, with a convergence criterion of 10^{-6}.

$$(3) \quad Wrec_{ij}^{(n)} = Wrec_{ij}^{(n-1)} \left(\frac{(E\ Cfix^T)_{ij}}{(Wrec^{(n-1)}\ Cfix\ Cfix^T)_{ij}} \right);\ f(Wrec, Cfix) = \frac{\|E - Wrec\ Cfix\|_F}{\sqrt{m\,n}}$$

$$(4) \quad Crec_{ij}^{(n)} = Crec_{ij}^{(n-1)} \left(\frac{(Wfix^T\ E)_{ij}}{(Wfix^T\ Wfix\ Crec^{(n-1)})_{ij}} \right);\ f(Wfix, Crec) = \frac{\|E - Wfix\ Crec\|_F}{\sqrt{m\,n}}$$

4 Inter-participant variability: randomized synergy ordering

In order to reconstruct C_{rec} of participant 1, a randomized 16 × NoSoA matrix W_{rand} was created. W_{rand} was reordered based on the CosSim similarities with synergy vectors W from participant 2 (…, 7). Next, we used the reordered W_{rand} matrix as an input in the recomputing algorithm. To reconstruct W_{rec} of participant 1, a randomized NoSoA × 600 matrix C_{rand} was created. Subsequently, C_{rand} was used to reconstruct W_{rec} of participant 2 (…, 7) utilising the respective EMG data. The synergies of the W_{rec} of participant 2 (…, 7) were compared with those of the original W of participant 2 (…, 7), with C_{rand} being reordered in accordance with the outcomes of this comparison. The reordered C_{rand} was then used to reconstruct W_{rec} of participant 1. This reordering procedure of randomized matrices served the purpose of comparing ordered synergies among participants. The matrices were sorted according to the synergy vectors, aligning with the original ordering of synergies which was also based on their W similarities.

5 Trick difficulty

In the main paper, we defined trick difficulty based on the required learning time to master the tricks properly and ranked the tricks as follows: easy – Ollie, medium – Kickflip, and hard – 360°-flip. Another possible approach to classify trick difficulties is the number of attempts needed for each trick to perform it successfully six times. Here, a lower number of attempts would indicate a lower difficulty. Therefore, an ANOVA was performed in a manner analogous to section 2.6.1 of the main paper to compare the number of attempts among the three tricks.

Although the ANOVA indicated significant differences ($p = 0.044$), post-hoc pairwise comparisons revealed that there were no significant differences among tricks ($p = 0.146 – 1$). However, the descriptive statistics indicate that the number of attempts for the Ollie (6.71±1.11) was lower than that for the Kickflip (12.43±4.04) and the 360°-flip (13.43±9.41). The number of attempts made by each participant for each trick is presented in Figure 2 of the main paper.

6 Figures

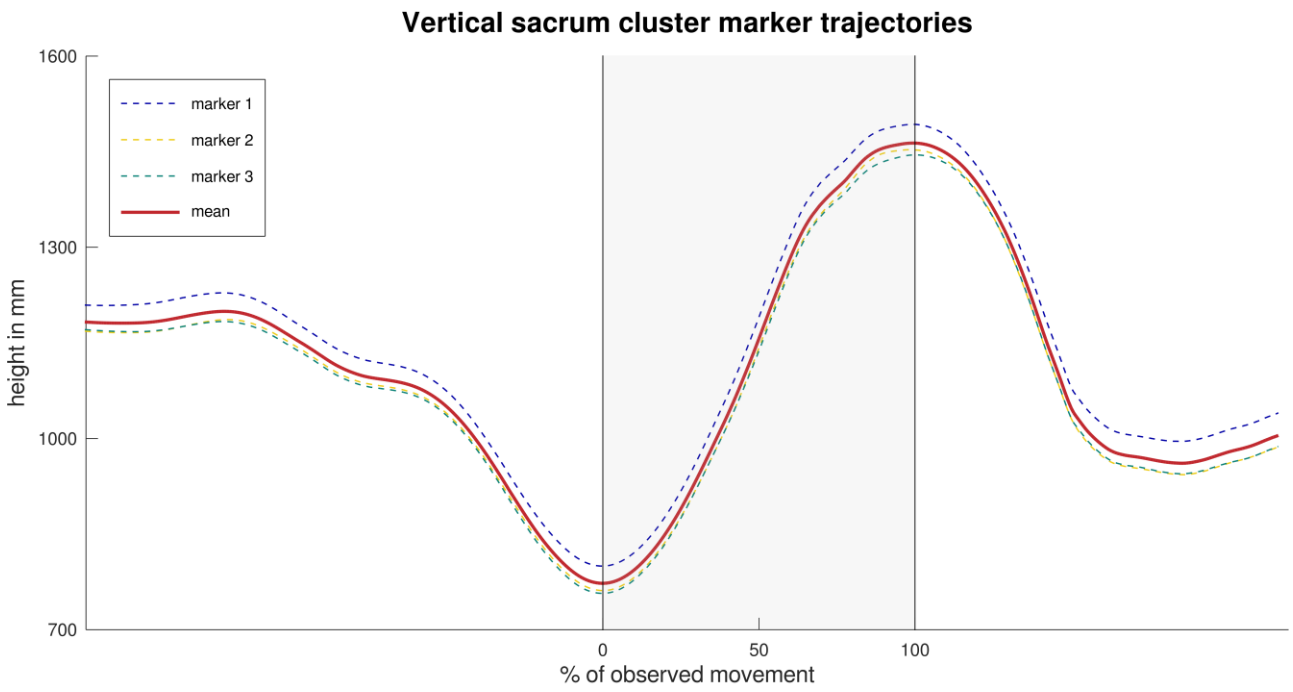

Figure S1: Example of the vertical sacrum cluster marker trajectories of an Ollie. The time interval between the lowest (0%) and highest (100%) point of the mean curve was analyzed (% of observed movement).

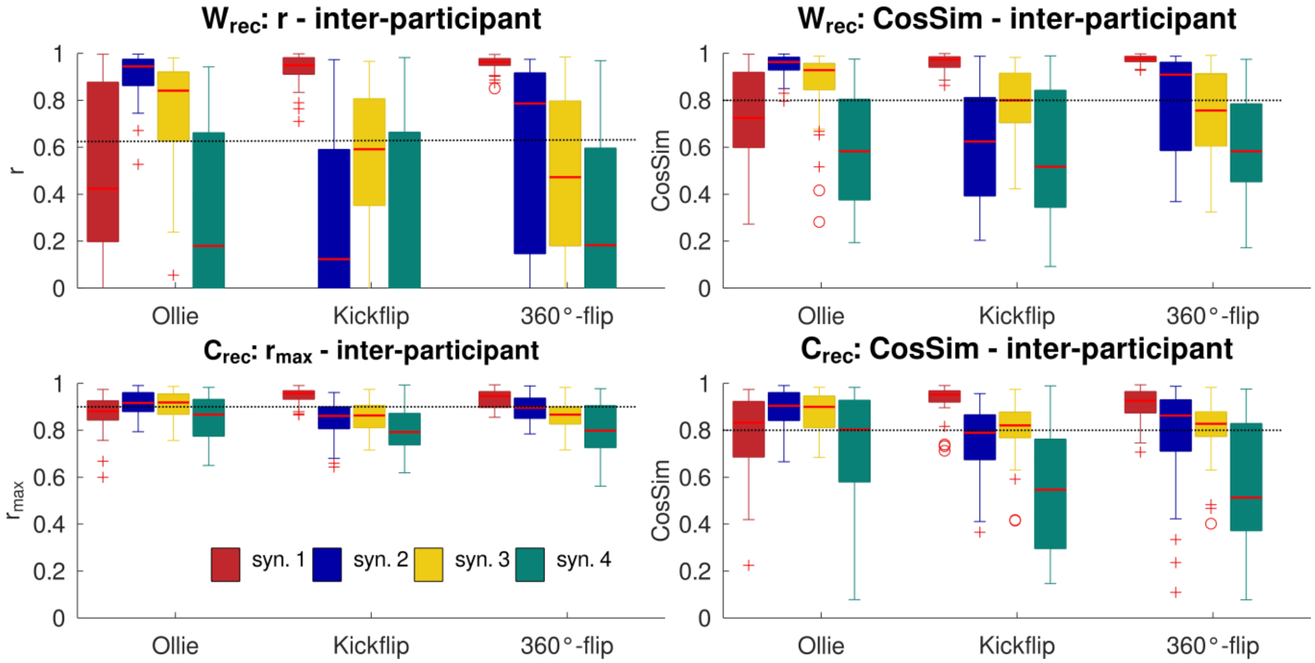

Figure S2: Boxplots showing the comparison values between reconstructed synergy vectors (W_{rec}, subplot a and b) versus original synergy vectors, and reconstructed activation coefficients (C_{rec}, subplot c and d) versus original activation coefficients. Synergies were reconstructed by synergies of the other participants for synergies (syn.) 1 to 4. Horizontal lines indicate chosen similarity thresholds: Pearson correlation coefficient (r) > 0.623, cross-correlation coefficient (r_{max}) > 0.9, cosine similarity (CosSim) > 0.8.

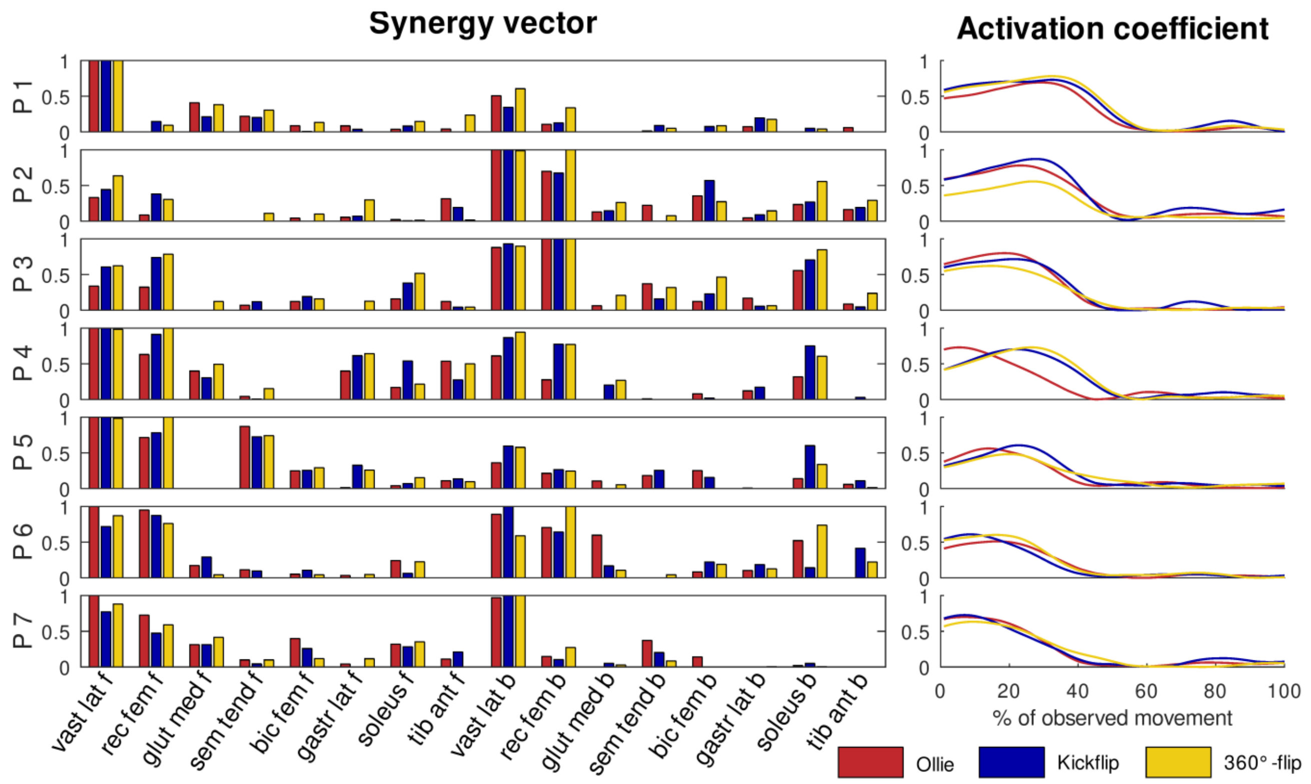

Figure S3: Synergy vectors and activation coefficients which were shared across all three tricks of all participants (P1…P7) based on the sharedOA method; y-ticks (0-1) are the muscle weightings (left subplots) or level of activation (right subplots) for synergy vectors or activation coefficients. Each waveform indicates the average activation coefficient across trials per trick. x-ticks indicate the muscles for the front (f, muscle 1-8) and back (b, muscle 9-16) leg. The time interval between the lowest (0%) and highest (100%) point of the sacrum cluster marker was analyzed (% of observed movement, see supplementary Figures S1).

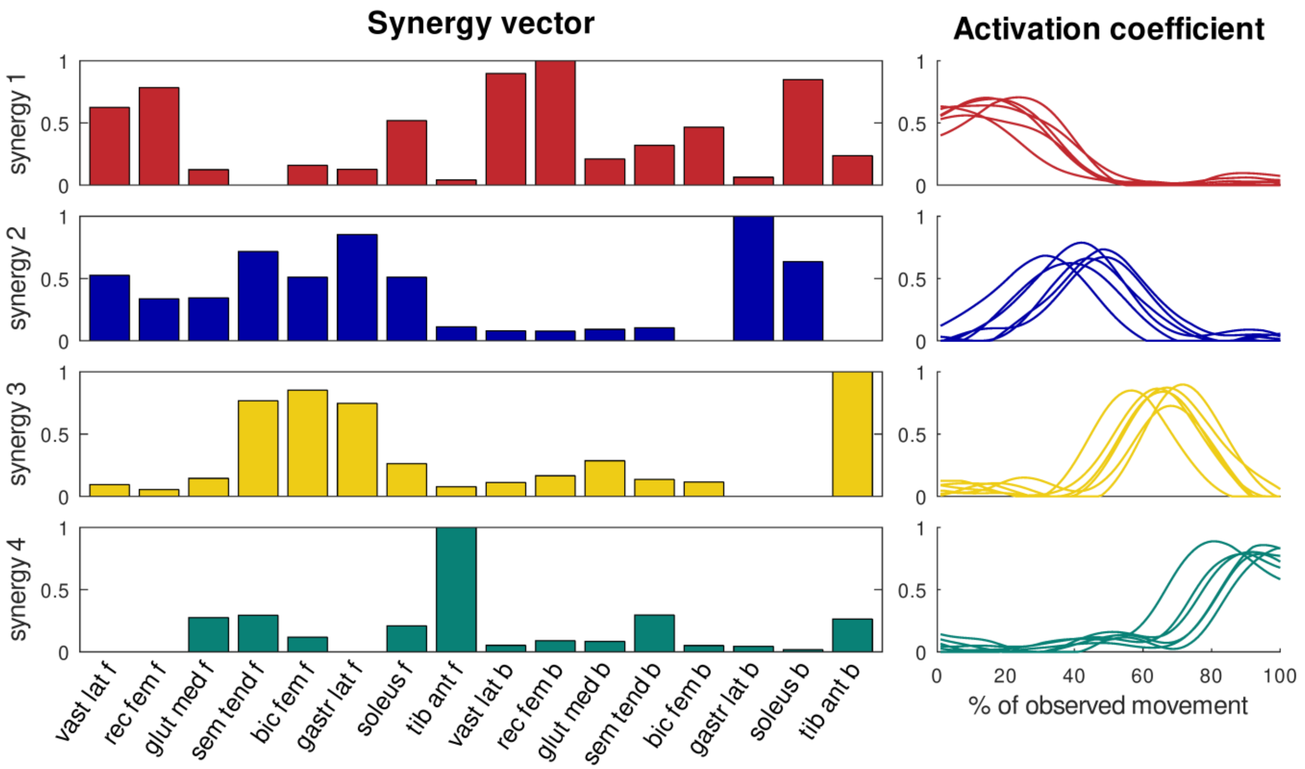

Figure S4: Example of all synergy vectors and activation coefficients for the Kickflip of one participant based on NoS and NoSOA (both 4 in this case); y-ticks (0-1) are the muscle weightings (left subplots) or level of activation (right subplots) for synergy vectors or activation coefficients. Each waveform indicates one trial. The time interval between the lowest (0%) and highest (100%) point of the sacrum cluster marker was analyzed (% of observed movement, see supplementary Figures S1). f = front leg; b = back leg.

7 Tables

Table S1: Number of synergies above our thresholds for reconstructed synergy vectors W and activation coefficients C of one trick, by the other two tricks, with original or random inputs for each participant; cosine similarity (CosSim) > 0.8 for recomputed W and C; Pearson's correlation coefficient (r) > 0.623 for recomputed W; cross-correlation coefficient (r_{max}) > 0.9 for recomputed C.

			original							random						
			participants													
Trick	rec. by		**1**	**2**	**3**	**4**	**5**	**6**	**7**	**1**	**2**	**3**	**4**	**5**	**6**	**7**
Ollie C	Kickflip W	>CosSim	4	2	3	2	3	3	2	3	1	3	3	2	1	0
		>r_{max}	4	2	3	3	3	3	2	2	0	2	2	2	0	0
Kickflip C	Ollie W	>CosSim	4	2	3	3	3	3	2	3	1	1	3	2	0	2
		>r_{max}	4	2	3	2	2	3	1	2	1	0	3	0	0	1
Ollie C	360°-flip W	>CosSim	3	4	3	2	4	2	3	3	2	1	1	0	1	2
		>r_{max}	3	4	3	3	3	2	2	1	1	1	1	0	0	0
360°-flip C	Ollie W	>CosSim	3	4	4	2	4	2	3	1	2	2	2	1	1	0
		>r_{max}	3	4	3	1	2	2	2	1	1	2	1	0	0	0
Kickflip C	360°-flip W	>CosSim	3	3	3	4	4	3	4	1	4	2	3	0	1	1
		>r_{max}	3	3	3	4	2	2	3	0	1	1	2	0	0	0
360°-flip C	Kickflip W	>CosSim	3	3	2	4	4	3	4	2	3	2	4	1	1	2
		>r_{max}	3	3	2	4	2	3	3	1	1	2	2	1	0	0
Ollie W	Kickflip C	>CosSim	4	2	3	3	4	3	3	2	2	2	2	2	2	1
		>r	4	2	3	3	4	3	3	0	1	2	0	1	2	1
Kickflip W	Ollie C	>CosSim	3	2	3	3	4	3	4	2	1	0	4	1	1	1
		>r	4	2	3	3	4	3	2	1	1	0	4	0	1	2
Ollie W	360°-flip C	>CosSim	3	4	4	2	4	3	2	3	2	1	2	0	0	1
		>r	3	4	3	1	4	3	2	2	2	0	1	0	0	1
360°-flip W	Ollie C	>CosSim	3	4	4	1	4	3	2	2	2	3	3	0	1	2
		>r	3	4	4	1	4	2	2	1	1	1	1	0	1	2
Kickflip W	360°-flip C	>CosSim	4	4	2	4	3	3	4	2	1	1	2	2	1	3
		>r	4	4	3	4	3	4	4	1	1	1	0	1	1	2
360°-flip W	Kickflip C	>CosSim	4	4	2	4	4	4	3	0	2	2	1	0	2	1
		>r	4	4	2	4	3	4	3	1	0	1	1	0	2	2

8 References

[1] P. Paatero and U. Tapper, "Positive matrix factorization: A non-negative factor model with optimal utilization of error estimates of data value" ," *Environmetrics,* vol. 5, pp. 111-126, 1994.

[2] H. S. Seung and D. D. Lee, "Learning the parts of objects by non-negative matrix factorization," *Nature,* vol. 401, p. 791, 1999, doi: 10.1038/44565.

[3] D. Lee and H. Seung, "Algorithms for Non-negative Matrix Factorization," *Adv. Neural Inform. Process. Syst.,* vol. 13, 02/10 2001.

[4] J. L. Allen, H. D. Carey, L. H. Ting, and A. Sawers, "Generalization of motor module recruitment across standing reactive balance and walking is associated with beam walking performance in young adults," *Gait Posture,* vol. 82, p. 247, 2020, doi: 10.1016/j.gaitpost.2020.09.016.

[5] F. O. Barroso *et al.*, "Shared muscle synergies in human walking and cycling," *J Neurophysiol,* vol. 112, p. 1998, 2014, doi: 10.1152/jn.00220.2014.

[6] N. A. Bianco, C. Patten, and B. J. Fregly, "Can Measured Synergy Excitations Accurately Construct Unmeasured Muscle Excitations?," (in eng), *J Biomech Eng,* vol. 140, no. 1, Jan 01 2018, doi: 10.1115/1.4038199.

[7] G. Boccia, C. Zoppirolli, L. Bortolan, F. Schena, and B. Pellegrini, "Shared and task-specific muscle synergies of Nordic walking and conventional walking," *Scand J Med Sci Sports,* vol. 28, p. 918, 2018, doi: 10.1111/sms.12992.

[8] G. Torres-Oviedo and L. H. Ting, "Subject-specific muscle synergies in human balance control are consistent across different biomechanical contexts," *J Neurophysiol,* vol. 103, p. 3098, 2010, doi: 10.1152/jn.00960.2009.

[9] M. M. Nazifi, H. U. Yoon, K. Beschorner, and P. Hur, "Shared and task-specific muscle synergies during normal walking and slipping," *Front Hum Neurosci,* vol. 11, p. 40, 2017, doi: 10.3389/fnhum.2017.00040.

[10] J. Kim and H. Park, "Toward Faster Nonnegative Matrix Factorization: A New Algorithm and Comparisons," presented at the Proceedings of the 2008 Eighth IEEE International Conference on Data Mining, 2008. [Online]. Available: https://doi.org/10.1109/ICDM.2008.149.

[11] F. Hug, N. A. Turpin, A. Guével, and S. Dorel, "Is interindividual variability of EMG patterns in trained cyclists related to different muscle synergies?," (in eng), *J Appl Physiol (1985),* vol. 108, no. 6, pp. 1727-36, Jun 2010, doi: 10.1152/japplphysiol.01305.2009.

[12] J. Frère and F. Hug, "Between-subject variability of muscle synergies during a complex motor skill," (in English), *Frontiers in Computational Neuroscience,* Original Research vol. 6, no. 99, 2012-December-28 2012, doi: 10.3389/fncom.2012.00099.

[13] C. L. Banks, M. M. Pai, T. E. McGuirk, B. J. Fregly, and C. Patten, "Methodological choices in muscle synergy analysis impact differentiation of physiological characteristics following stroke," *Front Comput Neurosci,* vol. 11, p. 78, 2017, doi: 10.3389/fncom.2017.00078.

[14] S. A. Safavynia and L. H. Ting, "Task-level feedback can explain temporal recruitment of spatially fixed muscle synergies throughout postural perturbations," *J Neurophysiol,* vol. 107, p. 177, 2012, doi: 10.1152/jn.00653.2011.

[15] M. Kristiansen, A. Samani, P. Madeleine, and E. A. Hansen, "Muscle synergies during bench press are reliable across days," *J Electromyogr Kinesiol,* vol. 30, p. 88, 2016, doi: 10.1016/j.jelekin.2016.06.004.

[16] F. Hug, N. A. Turpin, A. Couturier, and S. Dorel, "Consistency of muscle synergies during pedaling across different mechanical constraints," (in eng), *J Neurophysiol,* vol. 106, no. 1, pp. 91-103, Jul 2011, doi: 10.1152/jn.01096.2010.

[17] S. Muceli, A. T. Boye, A. D'Avella, and D. Farina, "Identifying representative synergy matrices for describing muscular activation patterns during multidirectional reaching in the horizontal plane," *J Neurophysiol,* vol. 103, p. 1542, 2010, doi: 10.1152/jn.00559.2009.

[18] L. Gizzi, J. F. Nielsen, F. Felici, Y. P. Ivanenko, and D. Farina, "Impulses of activation but not motor modules are preserved in the locomotion of subacute stroke patients," *J Neurophysiol,* vol. 106, p. 210, 2011, doi: 10.1152/jn.00727.2010.

[19] W. van den Hoorn, J. H. van Dieen, P. W. Hodges, and F. Hug, "Effect of acute noxious stimulation to the leg or back on muscle synergies during walking," *J Neurophysiol,* vol. 113, p. 54, 2015, doi: 10.1152/jn.00557.2014.
